# 

**Published:** 1853-06

**Authors:** 


### INDEX TO VOLUME NINTH.

☞ *For original articles look for the name of the author as well as for the title of the subject.*

For selected and editorial articles look for the subject alone.

**A** — Page.

Anæsthetics, use of, in rigidity of the perineum in first labor. By J. H. Beech, M. D., ........ 39
Ascites, treated by intra-peritoneal injections, ........ 41
Address to the Graduates in Medicine at the Univ. of Buffalo, April 28, 1853. By Frank H. Hamilton, M. D., ........ 65
Astley Cooper Prize, ........ 161
Annual Address before the N. Y. State Med. Society, by Dr. Alonzo Clark, ........ 176
Action of Medicines on the System. Headlands, ........ 188
An account of experiments and a post-mortem examination, ........ 183
Asphyxia from Chloroform, ........ 181
Am. Med. Association, Meeting of, .. 45
" " " its volumes of Transactions, ........ 222
" " " Doct. Page's Circular, ........ 241
" " " Sixth Annual Meeting of. By C. B. Coventry, M. D., ........ 129
" " " Seventh Annual Meeting, ........ 501
" " " Committees of, for 1854, ........ 160
Agassiz, Prof. L. Lectures before Buffalo Young Men's Association, 620
Anatomical Pursuits, ........ 320, 440
Albumen in Urine, ........ 486
Agriculture, New work on, ........ 127
Asylums for persons of unsound mind 254
Armour's Prize Essay, ........ 400

**B**

Beech, Dr. J. H., on anæsthetics in first labor, ........ 39
Bowditch, Henry I., on Diaphragmatic Hernia, ........1, 65

Page.

Bismuth in cholera infantum, ........ 179
Bite of a copperhead snake, ........ 44
Bronchophony and bronchial respiration, ........ 52
Blood Poisons, ........ 243
Botany, principles of, exemplified in the cryptogamia, ........ 251
Biological Society of New York, Dr. Dalton in, ........ 288
Buffalo Hospital of the Sisters of Charity, ........ 314
Buffalo Med. College, commencement of term, ........ 315
" " " condition of, ........ 365
" " " close of Lectures in, 629
" " " commencement exercises in, ........ 630
" " " summer course, ... 431
Births, Marriages and Deaths, ........ 369
Beaumont, death of, ........ 190
Buffalo, Monthly Record of Deaths in, ........602, 744
Butler, M. D., Wm. C., on Dysentery, 216
" on Veratrum Viride, ........ 719
" on Fractured clavicle, ........ 116
Budd on Diseases of the Liver, ........ 254
Ballou, Dr. N. E., on Schirrus of Stomach, ........ 340
Bellingham on Diseases of the Heart, 343

**C**

Coventry, Dr. Charles Brodhead, on Amer. Med. Association, ........ 129
" elected President of State Medical Society, ........ 628
Carpenter's Principles of Human Physiology, ........ 60
Cupping Apparatus, new, ........ 62
Chloroform in Labor, sanctioned by Royal example, ........ 58
" in Puerperal Convulsions, ........ 169
" Asphyxia from, ........ 181

PAGE.
Chloroform, Death from. By T. K. De Wolf, M. D., ... 385
" another, ... 612
" in Hooping Cough, ... 493
" in inducing narcosis from opium previously taken, ... 571
" Syncope from, ... 757
Caldwell, death of, ... 190
Chapman, " " ... 190
Corsets, history of, ... 233
Cucumber Ointment, note on, ... 298
Cancrum Oris, chlorate of potash in, 303
Colleges, Medical, ... 305
Cerebral Disease, statistics of, ... 308
Cartwright, Dr., and Mrs. Willard, ... 310
Cholera, ... 375
" epidemic, ... 555
Climatic conditions of 1853, ... 402
Condie on Diseases of children, ... 445
Coffee, on the effect of, ... 492
Cerebral affections of children. By S. B. Hunt, M. D., ... 519
" circulation, clinical remarks on. By Chas. A. Lee, M. D., ... 707
Correction, ... 746
Contributors to Buff. Med. Jour. for 10th vol., ... 758

D

Dysentery, clinical report on. By Austin Flint, M. D., ... 108, 136, 193
" Supplement to, ... 257
" Remarks on. By Wm. C. Butler, M. D., ... 216
" Cold water in, ... 239
" Opium in, ... 255
" Salines in, ... 503
Dislocation of the Shoulder. By Eli Hurd, M. D., ... 119
" of the Hip, ... 368
Diabetes, ... 123
Dwight, death of Dr., ... 126
Doctors, ... 179
Dewey, M. D., D. C., rectum, imperforate, ... 214
Drake, death of, ... 242
Dowler, Dr. Bennett, ... 256
Dalton on Fishes of the Mammoth Cave, ... 288
" Prof. J. C., notice of, ... 447
" Vivisections at Buffalo Med. College, ... 622
" Testimonial to, ... 636
Distribution of Arteries, unusual, ... 368
Draper's Introductory, ... 376
De Wolf, Dr. T. K. Case of death from chloroform, ... 385
De Boismont on Hallucinations, ... 442
Dunglison's Materia Medica, ... 472
"De omnibus rebus et quibusdam aliis." By Rusticus, ... 526
Dublin Medical Press *vs.* Dr. Flint's Prize Essay, ... 557

PAGE.
Dissections, legalized, ... 568
Death from plugging of pulmonary veins, ... 617
Dissection Bill and N. Y. Daily Times 633
Divinity *vs.* Physic, ... 746

E

Errata, ... 45, 564
Editorial change, ... 61
Erie County Medical Society, ... 64, 562
Erysipelas, ... 121
Eve, Prof. Paul F., on Stricture of the Urethra, ... 157
" removal of a Nail from the Lungs by, ... 316
Ergot in Retention of Urine, ... 171
" substitute for, ... 181
Experiments and post-mortem examination, ... 183
Epilepsy, ... 297
Erysipelas, an analysis of the treatment of, by S. B. Hunt, ... 462
Epidemic in New Orleans, ... 541
Education, report to the committee on Medical, ... 598
Electro-Biology, ... 607

F

Fergusson's System of Practical Surgery, ... 57
Flint, M. D., Austin, clinical report on Dysentery, ... 108, 136, 193
" clinical report, supplement to, 257
" on Pericarditis masked by delirium, ... 449
" analysis of twenty-one cases of Articular Rheumatism, ... 577
" departure of, for Europe, ... 620
Fracture of Clavicle. By Dr. Wm. C. Butler, ... 116
Fracture Tables, Prof. Hamilton's, ... 187
Fracture of Radius and Ulna, splint for, by E. P. Smith, M. D, ... 225
Fracture of Clavicle, new splint for, ... 242
Fractures, new dressing for, ... 369
Fracture of Patella, adhesive plaster in, ... 545
Felt Splints. By F. H. Hamilton, M D., ... 399
Females, Medical education of, ... 427
Faults of medical writers, ... 475
Fistula Lachrymalis, ... 502

G

Gross on Malignant Diseases, ... 162
Gluge, Gottlieb. Atlas of Pathological Histology, ... 252
Galactagogues, ... 310
Geneva Medical College, ... 316
Germain's Speech on the Dissection Bill, ... 736
Guano in skin diseases, ... 757

H — Page.

Hamilton, Professor Frank H. Address to Graduates, ... 95
" Fracture Tables, ... 187
" Felt Splints, ... 399
Hernia, Diaphragmatic. Dr. H. I. Bowditch on, ... 1, 65
" mention of, ... 64
Heart Clot, sudden death from, ... 58
Hurd, M. D., Eli, on compound dislocation of shoulder, ... 119
Hall, Dr. Marshall, reception at Buffalo, ... 174
" mention of, ... 383
" on tracheotomy in Puerperal Convulsions, ... 705
Hernia, committee on *vs.* Dr. Heaton, 185
Henle's General Pathology, ... 190
Humulus Lupulus, antiperiodic powers of, ... 301
Hay Asthma, ... 358
Human Heads and their covering, ... 357
Hygrometric Observations, ... 441
Howard, Prof. R. L., death of, ... 634
Hunt, M. D., Sanford B. An analysis of sixty-seven cases of Inversio Uteri, ... 321
" A case of Pleuro-Pneumonia with Pleuritic Abscess, ... 389
" An analysis of the treatment of Erysipelas, ... 462
" On Iodide of Potassium in cerebral effusions, ... 519
Homœopathy fairly represented. By Wm. Henderson, M. D., ... 743

I

Iodide of Potassium, ... 480
Impalement on a pitchfork handle, ... 489
Infection and contagion, ... 552

J

Jenner, monument to, ... 256
Jamaica, Fevers of, ... 420

K

Knight's Introductory, ... 573

L

Lead Poisoning, elimination of, by Iodid. Potassii, ... 167
" Palsy treated by Iodid. Potassii. By J. M. Newman, M. D., ... 387
Leaden Pipes, remarks on, ... 361
Labor, premature, induction of, in obstinate vomiting of pregnancy, . 234
Lee on Homœopathy, ... 573
" on cerebral circulation, ... 707
Lord, Rev. Dr. John C., on Medical Science and Materialism, ... 637

M

Medical examinations in London, ... 63
Malaria, ... 123

Page.

Malpractice, ... 167, 556
Microscopic Preparations, ... 230
Miller's Practice of Surgery, ... 253
Meigs' Practical Treatise on Diseases of Children, ... 254
Medical Journals, ... 335
Medical Colleges, opening of, ... 384
" " ... 550
Medical Research, Thoughts on, by "Rusticus," ... 393
Medical Theories, Thoughts on, by "Rusticus," ... 469
Meacham, Dr. Wm. G., on vaccination, 457

N

Newman, Dr. James M., on American Med. Association ... 225
" on Lead Palsy, ... 387
" Monthly Record of Deaths in the city of Buffalo, ... 603, 744
Nervous Sympathy, thoughts on its rationale, ... 436

O

Obituaries of Dr. W. C. Dwight, ... 126
" Dr. Geo. O. J. Du Relle, 126
" Dr. Joseph Peabody, ... 126
" Dr. Chapman, ... 190
" Dr. Wm. Beaumont, ... 191
" Dr. Charles Caldwell, ... 191
" M. Orfila, ... 227
" Dr. R. L. Howard, ... 634
Opium Eating, ... 4[illegible]2

P

Pneumonia, blood-letting in, ... 44
Pleuro-pneumonia, S. B. Hunt, M. D., 389
Pharyngitis, chronic, ... 163
Pisciculture, ... 236
Phthisis Pulmonalis, ... 298
Puerperal Peritonitis, Prof. James P. White on, ... 461
Peritonitis, chronic, modified by scrofula. By H. M. T. Smith, M. D., 531
Puerperal Fever, ... 304
" Convulsions, tracheotomy in. By Dr. Marshall Hall, ... 705
Placenta, development of, in Fallopian Tubes, ... 344
Paracentesis Thoracis, ... 349
Potassæ Liquor, effects of, on the urine, ... 363
Purpura Hæmorrhagica, gallic acid in, ... 423
Pericarditis marked by delirium. By Austin Flint, M. D., ... 449
Pharmacy, contributions to, ... 476
Platform, medical, ... 499
Paget's Surgical Pathology, ... 532
Patella, fracture of ... 545
Palmer's Introductory Lecture, ... 572
Promotion of Medical Science, Mr. Germain's speech on, ... 736

**Q** PAGE.

Quinine in Rheumatism, ........... 122
" in Continued Fever, ....... 377

**R**

Rochester, Prof. Thos. F., appointment of, .................198, 241
" Address to Graduates, ....... 641
Rectum, imperforate, Dr. D.C. Dewey on, ........................... 214
Rheumatism treated by Quinine, ... 122
" " " Veratrine, . 235
" expectant treatment of.... 503
" analysis of twenty-one cases, by A. Flint, M. D., ........ 578
Rattlesnake Bite, treatment of, by E. Stanley, M. D., ............... 464
Ricord and Hunter on Venereal Disease, ........................... 446
Re-vaccination, ................. 303
"Rusticus," on Medical Research,.. 393
" " " Theories, .. 469
" " Veratrum Viride, .. 595
" De omnibus et quibusdam aliis 526
" Thoughts on Transfusion,.... 729
Ricord on Syphilis, .............. 243
Report of the Committee on Medical Education, .................... 598
Robinson, Dr. C. D., on Typhoid Fever, ............................ 513

**S**

Shoulder, dislocation of, by Eli Hurd, M. D., ........................ 119
Simpson's contributions to obstetrics, 185
Smith, Ellery P. New splint, ...... 225
Strychnia in impaired spinal energy, 241
Statistics, ...................... 247
Small-pox, ...............290, 494, 632
Scarlatina, ...................... 302
Stomatitis, ...................... 303
Schirrus of Stomach, by N. E. Ballou, M. D., ...................... 340
Stricture, Benique's method of treating, ........................... 369
Spiritual manifestations, .......... 372
Sims on Vesico-vaginal Fistula, .... 401
Stanley, Dr. E., on Rattlesnake bite, 464
Specialties, ..................... 496
Sympathetic Nerve and its functions 508
Schiedam Schnapps, .............. 511
Spooneyism, .................... 574

PAGE.

Smith, Dr. H. M. T., on chronic peritonitis, ....................... 531
Sold out, ....................... 575
State Medical Society, ........... 626
Smith, Dr. J. V. C., elected Mayor of Boston, ....................... 635

**T**

Tracheotomy in Epilepsy and Apoplexy, ........................ 51
Tilt's Elements of Health, ........ 251
Tic Doloreux, ................... 302
Table Turning, .................. 299
Typhoid Fever, .................. 304
" " and Typhoidism,..... 433
" " treatment of, by C. D. Robinson, M. D., ............... 513
Tape Worm, ..................... 362
Transactions of Penn. State Society, 383
" State Medical Society, 1854,.. 753
" Amer. Med. Association,..... 734
Tetanus, ....................... 424
Talpa, ......................... 444
Transfusion of Blood, Thoughts on. By Rusticus, ................. 729

**U**

Unity of Disease, ................ 249
Uterus, inversion of, by S. B. Hunt, M. D., ....................... 321
Urine. Its condition in Typhoid Fever, ........................ 418

**V**

Veratrum Viride, ................ 244
" " By Wm. C. Butler, M. D., ....................... 719
Volitional control of involuntary muscles, ...................... 434
Vaccination, W. G. Meacham on,.... 457

**W**

Williams' Principles of Medicine,... 255
Walton's Operative Ophthalmic Surgery, ......................... 316
White, Prof. James P., on Puerperal Peritonitis, ................... 461

**Y**

Yellow Fever in N. Orleans, 256, 309, 751
Yankeedom Outyankied, .......... 755

### *Erie County Medical Society.*

The Semi annual Meeting of this Society, will be held in this city on the 14th of June, instant, at 10½ o'clock, A. M., at American Hall.

JAMES M. NEWMAN,
*Recording Secretary.*

BUFFALO, *June* 1, 1853.

In calling attention to the above notice we would remind members of the Society, that the plan pursued at the last meeting was to continue the morning session till all the business before the meeting is concluded, and adjourn to a dinner provided by the physicians of Buffalo. It is expected that the same course will be adopted at the meeting in June.

We perceive, by some of our exchanges, that the annual vol. of Transactions of the New York State Society has been issued. This is the only evidence of the fact that we have yet received.

---

Dr. Hurd's article on Compound Dislocation of the Shoulder, was received too late for insertion in this No. It will appear in our next issue.

